# Complete genome sequence of *Priestia aryabhattai* KC-6A, isolated from peatland of northern Japan

**DOI:** 10.1128/mra.00639-25

**Published:** 2025-07-31

**Authors:** Tomoyoshi Hashimoto, Takeshi Shoda, Midori Sakoda, Megumi Ito, Mai Iwamoto-Shizukuda, Daisuke Fukahori, Yasuo Ikegashira, Tomoyasu Nishizawa

**Affiliations:** 1 Tsukuba Research Institute, Katakura & Co-op Agri Corporation, Tsuchiura, Ibaraki, Japan; 2 Graduate School of Agriculture, Ibaraki University, Ami, Ibaraki, Japan; 3 Green-Bio Technology Research Center, Ibaraki University, Ami, Ibaraki, Japan; Indiana University Bloomington, Bloomington, Indiana, USA

**Keywords:** *Priestia*, peatland, gram-positive bacteria, genome sequence

## Abstract

The genus *Priestia*, formerly referred to as *Bacillus*, is capable of growing under abiotic stresses in the ecosystem. We now report the complete genome sequence of *Priestia aryabhattai* KC-6A, which was obtained from culture soil utilizing peatland as an agricultural material.

## ANNOUNCEMENT

Peatlands are inherently unsuitable for agriculture because of their high acidity, poor inorganic content, and susceptibility to decomposition and nitrogen accumulation during high summer temperatures. However, bioprospecting for beneficial bacteria associated with decomposing soil organic matter offers a promising utilization of peat soil (1).

The indigenous bacteria in peatland located in Hokkaido (43.22°N, 141.51°E), Japan were cultured and grown on a carboxymethylcellulose agar plate (2) using 10-fold serial dilutions in sterile distilled water of each soil (up to 10^6^ dilutions) at 30°C for 7 days. The 16S rRNA sequence of the restreaked isolate, strain KC-6A (accession number, PV436949), was phylogenetically identified in 99.9% of the *Priestia aryabhattai* B8W22^T^ (3). Genomic DNA (gDNA) was extracted from colonies of KC-6A cells grown in R2A broth (FUJIFILM Wako Pure Chemical Corporation, Japan) at 30°C for 24 h with a DNeasy Power Soil Kit (Qiagen, Germany) according to the manufacturer’s protocol. A 150 bp paired-end library of KC-6A gDNA was prepared using an MGIEasy PCR-free DNA library prep set (MGI Tech., China) and sequenced on the DNBSEQ-G400 platform (MGI) to generate 1.29 Gb of short reads (4,304,989 raw read pairs). Adapter-sequence removal and read filtering were performed using Cutadapt v4.1 (“--overlap 10,” “--minimum-length 51,” and “--quality-cutoff 20”) (4). A nanopore sequencing library was prepared using the same gDNA without shearing or sorting the DNA by size utilizing a Native Barcoding Kit (SQK-NBD114.24; Oxford Nanopore Technologies, Oxford, UK) and sequenced on a PromethION platform (R10.4.1; ONT) to generate 1.62 Gb of long reads (307,362 raw reads). Base calling and adapter sequence removal were performed using Guppy v6.5 with SUP model (5) and Porechop v0.2.4 (6), respectively. High-quality reads were selected using NanoFilt v2.7.1 (“-l 1000” and “-q 12”) (7). Finally, 4,304,968 paired-end short reads (2 × 150 bp) and 208,830 long reads (average length, 7,030 bp; *N*_50_ value, 10,143) were obtained from the MGISEQ and Nanopore libraries, respectively. The Nanopore reads were assembled using Trycycler v0.5.3 (8) to produce five circular contigs by specifying *dnaA* for the rotation of the genome, which were subsequently polished using Medaka v1.7.2. The Burrows–Wheeler Aligner v0.7.17 (9) and Pilon v1.23 (10) were used with the short-read data set. The genome sequences were annotated using PGAP v6.9 (release 31) (11) and discriminated between chromosomes and plasmids by RFPlasmid v.1.0 (12). The genome completeness was identified using CheckM v1.2.3 (13), and the completed genome was identified phylogenomically using JSpeciesWS V4.2.1 (14). Default parameters were used except where otherwise noted.

The 5,277,486 bp genome (261.4× coverage) with a GC content of 35.1% consists of a circular chromosome and four circular plasmids. The KC-6A genomic features are shown in Table 1. To assess the species definition, whole-genome average nucleotide identity was examined using draft genomic sequences of *P. aryabhattai* B8W22^T^ (NCBI accession number, GCF_000956595.1), revealing 95.7% sequence similarity with strain KC-6A.

**TABLE 1:** Genome features of *Priestia aryabhattai* KC-6A

Genome features	Contig1	Contig2	Contig3	Contig4	Contig5
Total length (bp)	5,039,259	145,298	76,383	9,301	7,245
Alias	Chromosome	Plasmid	Plasmid	Plasmid	Plasmid
Form	Circular	Circular	Circular	Circular	Circular
GC content (%)	38.5	33.5	36.5	34.0	33.0
No. of CDSs	5,095	159	59	9	9
No. of rRNAs	39	0	3	0	0
No. of tRNAs	113	1	17	0	1
No. of tmRNAs	1	0	0	0	0
Completeness (%)	99.27				

## Data Availability

The genome sequences of KC-6A were deposited in DDBJ/ENA/NCBI under SRA numbers SRR31691750 and SRR31691751, and under accession numbers CP174608, CP174609, CP174610, CP174611, and CP174612.
